# Impact of Covid-19 Restrictions on Individual Placement and Support Service Delivery in Northern Norway

**DOI:** 10.1007/s40737-022-00304-5

**Published:** 2022-09-17

**Authors:** Sina Wittlund, Daniil Butenko, Oda Lekve Brandseth, Beate Brinchmann, Eóin Killackey, David McDaid, Miles Rinaldi, Arnstein Mykletun

**Affiliations:** 1 Centre for Work and Mental Health, Nordland Hospital Trust, Bodø, Norway; 2 Department of Community Medicine, UiT – The Arctic University of Norway, Tromsø, Norway; 3 Department of Clinical Medicine, The University of Bergen, Bergen, Norway; 4 Centre for Research and Education in Forensic Psychiatry, Haukeland University Hospital, Bergen, Norway; 5 Orygen, Melbourne, Australia Centre for Youth Mental Health, Melbourne, Australia; 6 The University of Melbourne, Melbourne, Australia; 7 Department of Health Policy, Care Policy and Evaluation Centre, London School of Economics and Political Science, London, UK; 8 South West London and St George’s Mental Health NHS Trust, London, UK; 9 Division for Health Sciences, Norwegian Institute of Public Health, Oslo, Norway

**Keywords:** Individual placement and support, Employment specialist, Covid-19 restrictions, Service delivery, Work, Mental illness

## Abstract

Individual Placement and Support (IPS) is an evidence-based supported employment program that helps people with severe mental illness to achieve steady, meaningful employment in competitive mainstream jobs. The purpose of this study is to investigate the impact of Covid-19 restrictions on IPS service delivery in Northern Norway between March and October 2020. In Norway, IPS is in the early stages of full-scale implementation and is therefore potentially sensitive to external stressors such as the Covid-19 pandemic. In October 2020 we conducted a retrospective, cross-sectional survey with IPS employment specialists in Northern Norway (n = 25). The purpose was to collect information about how Covid-19 restrictions between March and October 2020 impacted their ability to deliver IPS services. As a result of Covid-19 restrictions, more than half the employment specialists were reassigned to other roles or non-IPS related work tasks. They also reported less collaborative engagement with clinical teams and employers. 69 (20.4%) of IPS users supported by employment specialists gained employment after the Covid-19 restrictions were introduced and 82.8% of unemployed IPS users continued to seek competitive employment despite Covid-19 restrictions. Covid-19 restrictions appear to have created obstacles for IPS service delivery in Northern Norway and have negatively impacted the employment specialists' collaborative engagement with clinical teams. However, IPS employment specialists have shown strong capabilities in overcoming these challenges and services users have remained motivated to seek employment during the pandemic.

## Introduction

Individual Placement and Support (IPS) is an evidence-based supported employment intervention which can aid people with severe mental illness along the road to recovery (Bond, 2004). IPS provides individualised job development and the support required to help people with severe mental illness achieve steady, meaningful employment in competitive mainstream jobs (Bond, 2004). Employment specialists are an integral part of IPS service delivery. They provide a link between employment and specialist mental health services, thereby assisting people to find and maintain jobs of their choice, as well as provide support if jobs do not work out (Drake et al., 2012).

IPS is internationally recognised as being the most efficacious way of supporting people with severe mental illness into competitive employment (Brinchmann et al., 2020). Brinchmann et al. (2020) conducted a meta-analysis of IPS internationally and found that IPS is not moderated by GDP, regulation of temporary employment, unemployment rates for those with low education, generosity of disability benefits or type of integration policies. However, it has not been established if this finding holds true during the Covid-19 restrictions as the pandemic has created an unprecedented public health crisis (WHO, 2021).

### Mental Health Impacts of the Covid-19 Lockdowns

Despite concerns that Covid-19 lockdowns have negative repercussions for mental health, a recent meta-analysis of longitudinal studies during the pandemic finds that lockdowns had only a selective and modest impact on mental health indicators for anxiety and depression. This review also finds that lockdowns had no effect on positive psychological functioning, mental wellbeing or life satisfaction and did not negatively influence perceptions of loneliness or social support (Prati & Mancini, 2021).

There is however conflicting evidence in the literature addressing pandemic consequences for individuals with severe mental illnesses (SMIs) such as schizophrenia, bipolar disorder and other debilitating affective disorders. Van Rheenen et al. (2020) and González-Blanco et al. (2020) find that depression, anxiety and stress increased among people with SMI, while Muruganandam et al. (2020) reports that Covid-19 lockdowns were associated with re-emergence of psychiatric symptoms. Individuals with SMIs may also have higher rates of pandemic induced loneliness compared to the general population (Heron et al., 2022). On the contrary, Pinkham et al. (2020) provide evidence that individuals with pre-existing SMIs adapted well to the uncertainty and unpredictability accompanying the pandemic and that their day-to-day mental health remained stable. Somewhat unexpectedly, many study participants also reported a significant improvement in well-being post-pandemic onset (Pinkham et al., 2020). Furthermore, evidence from European studies to date suggests that the pandemic has not detrimentally impacted key indicators of population mental health such as non-fatal deliberate self-harm and completed suicide (RESPOND Project, 2021).

There is also evidence that the lockdowns have beneficial effects for mental health and wellbeing. Many individuals report the favourable impacts of home office arrangements such as saving the time and money of commuting, greater flexibility, more quality time with family, increased focus on fitness and improved work-life balance (Green et al., 2020). Lockdowns have also pioneered possibilities for a permanent shift to remote work, allowing workers to maintain these newly discovered benefits (RESPOND Project, 2021; Green et al., 2020).

### Mental Health Services During the Covid-19 Pandemic

#### Demand for Mental Health Services

Across Europe studies generally report decreased uptake of mental health services until November 2021 (RESPOND Project, 2021). This study was conducted in Norway in October 2020 and falls within this decreased uptake period.

#### Mental Health Service Adaptation

Mental health services which require extensive social interaction with individuals and others working outside inpatient wards and community teams are especially vulnerable to external stressors such as the Covid-19 pandemic. The Covid-19 restrictions have disrupted the traditional approach of delivering health care via face-to-face interaction and prompted a surge in telemedicine usage (O'Brien & McNicholas, 2020). There has been an explosion in telepsychiatry, a subset of telemedicine, which involves remote delivery of mental health services (i.e. psychiatric assessments, therapy, medication management etc.) via telecommunication platforms (O'Brien & McNicholas, 2020; Shore, 2020). Certain mental health services such as pharmacotherapy are relatively easy to manage remotely whereas other areas of mental health management require more intensive one-on-one, in person contact.

## Background Context

### Evolution of IPS in Norway

Traditionally, vocational rehabilitation services in Norway have followed a train-then-place approach. In the early 1990’s supported employment was introduced, spawning a range of new innovations aimed at increasing labour force participation (Sveinsdottir et al., 2020). More recent developments include a shift toward evidence-based IPS (Sveinsdottir et al., 2020), and results from a Norwegian RCT found that IPS improves both work- and health-related outcomes for people with moderate to severe mental illness (Reme et al., 2019).

Despite overwhelming RCT evidence for the efficacy of IPS there is still a lack of evidence regarding the effectiveness of IPS and the societal consequences of large-scale implementation (Boardman & Rinaldi, 2013). IPS services in Norway are provided through collaboration between two different governmental bodies, The Norwegian Labour and Welfare Administration (NAV) as a public employment service and health services (OECD 2013). Health services are represented both by primary mental health care at municipality level and secondary, specialist psychiatric care. Several government policies have both recommended and supported the scale up of IPS services across Norway (The Norwegian Directorate of Health, 2016; Norwegian Ministry of Labour and Social Affairs, 2017).

Several interconnected challenges make IPS in Norway vulnerable to external stressors such as the Covid-19 pandemic. IPS is integrated into the work of mental health services and is vulnerable to Covid-19 restrictions due to the need for extensive social interaction between IPS employment specialists, IPS users, clinical teams and employers. IPS employment specialists in Norway are employed by the welfare sector (NAV) but are expected to be integrated within mental health services. However, employment specialists reported that the challenges of intersectoral coordination create barriers for their integration into clinical mental health teams (Moe et al., 2021) and it is plausible that Covid-19 social distancing and stay-at-home policies may have further exacerbated integration difficulties. Norway is still in the early stages of full-scale IPS implementation with IPS services based on funding for temporary project positions. The majority of IPS employment specialists have been in the role for less than one year and many have a profession that they can return to if the employment specialist role does not work out. Furthermore, the employment specialist role is largely dependent on physical meetings with IPS users and direct collaboration with employers in the open job market. Thus, social distancing restrictions may detrimentally impact the employment specialists ability to deliver IPS services.

### IPS in Northern Norway

Northern Norway consists of two counties (Nordland, and Troms and Finnmark). The region is primarily rural, characterised by long distances between settlements. As a result, employment specialists in Northern Norway often need to make long and time-consuming commutes through sparsely populated areas in order to deliver IPS services. There are only two towns (Bodø and Tromsø) with a population over 50,000 inhabitants. The management of the IPS services are decentralised at each local NAV office, although training, supervision and implementation support is given from a central hub with a regional mandate (Moe et al., 2021; IPSNOR, 2018). At the time of this study, 45 employment specialists were employed full-time across 14 sites in Northern Norway.

### Time Frame of Covid Restrictions in Norway

In March 2020 the Norwegian government implemented a range of measures to mitigate the spread of coronavirus. This included social distancing and closures of businesses. Covid-19 restrictions continued with varying degrees of severity throughout 2020 and the Norwegian government also strongly recommended stricter local measures that municipalities with a high infection level should consider implementing. It is important to note that for most of the time period covered in the survey, the stringency of Covid-19 restrictions, the incidence rate of Covid-19 and hospitalisations due to Covid-19 were relatively low (Statistics Norway, 2021a).

### Labour market consequences of Covid-19 in Norway

In Norway, the number of job vacancies plummeted in the second quarter of 2020 but recovered rapidly in the following quarter (Statistics Norway, 2021a). Employment levels (Statistics Norway, 2021b) followed similar trends to other European countries (RESPOND Project, 2021), contracting abruptly in the second quarter of 2020 before spiking briefly in the following quarter, then declining again during winter 2020–2021 when Covid-19 rates rose sharply (Statistics Norway, 2021b). Thereafter, employment levels in Norway rebounded strongly (Statistics Norway, 2021b). Robust social welfare and economic Covid-19 protection strategies in Norway, such as tax exemptions and business compensation measures (National Insurance Act, 2020a, 2020b, 2020c; Norwegian Tax Administration, 2021) may have helped reduce labour market consequences of the pandemic.

### Furlough Scheme in Norway

Under normal circumstances (pre-pandemic), furloughed workers in Norway are entitled to an unemployment benefit from NAV for a maximum duration of 26 weeks, covering 62.4% of their regular salary (National Insurance Act 1988; NAV, 2020). Eligibility for unemployment benefits is based on previous earnings and duration of employment (NAV, 2020). In March 2020, in order to alleviate the negative effects of the Covid-19 crisis on employees, the government temporarily relaxed eligibility requirements and increased the generosity of unemployment benefits (National Insurance Act, 2020a). Furthermore, in November 2020 the government temporarily extended the period furloughed workers could receive an unemployment benefit from 26 to 49 weeks (National Insurance Act, 2020b).

### Aim

This study aims to investigate how the Covid-19 restrictions that were in place between March and October 2020 impacted the IPS service delivery in Northern Norway.

## Methods

### Study Design

In October 2020 we conducted a retrospective cross-sectional survey with IPS employment specialists in Northern Norway. The purpose was to collect information about how Covid-19 restrictions between March and October 2020 impacted their ability to deliver IPS services. The survey was administered using the online platform SurveyMonkey. The survey was developed by author MR in English and adapted by AM, DB and MR for a Norwegian context. The survey was translated into Norwegian and piloted prior to use. The survey had 32 items and the majority of items required either tick-box or used Likert scale responses.

### Sample

Initially, 40 of the 45 (88.9%) of the employment specialists in Northern Norway consented to be surveyed. The Covid-survey response rate was 62.5% of those who initially consented to be surveyed (n = 25). Demographic characteristics are summarised in Table 1.

**Table 1:** Demographic variables

Number of participants	n = 25
Age (mean, SD, range)	Mean = 40.5; SD = 7.7; min. = 26, max. = 55
Female gender n (%)	n = 17 (68.0%)
Time in IPS employment specialist role (mean, SD, range)	Mean = 8.2 (months); SD = 7.0; R = 0.0–36.0

### Statistical Methods

For paired nominal data the McNemar change test was used to determine if there were significant differences in how employment specialists communicated with IPS users pre and post Covid-19 restrictions. We used five-point likert scales to collect data on employment specialists' self-reported collaboration with the clinical teams and employers, as well as the impact of Covid-19 restrictions on the mental health and well-being of IPS users on their lists. A score of 1 indicated much less than usual and a score of 5 specified much more than usual. One sample *t*-tests with a test-value of 3 (same as usual) were used to ascertain if the Covid-19 restrictions significantly impacted these collaborations.

## Results

In March 2020, as a result of resource shifting due to the Covid-19 response, 52% of employment specialists reported that they were reassigned to other roles or received extra, non-IPS related work tasks. The employment specialists reported that they had a total of 338 IPS users on their lists between March and October 2020. Sixty-nine (20.4%) of the 338 IPS users gained employment after the Covid-19 restrictions were introduced in March 2020. 49.7% of IPS users were unemployed and jobseekers, 10.4% were unemployed and not looking for work due to the Covid-19 restrictions, 1.8% were on sick leave, 12.8% were temporarily laid off (furlough) and 4.7% lost contact.

One-sample *t*-tests (Table 2) showed that employment specialists perceived that they had significantly less collaboration than usual with both the clinical teams and with employers after the Covid-19 restrictions were introduced (*p* < 0.001). The employment specialists also reported that IPS users on their list experienced more than usual variation in their mental health (*p* < 0.001) and were more worried (*p* < 0.001) and socially isolated (*p* < 0.001). In addition, the employment specialists perceived that IPS users required more support than usual from both the employment specialists (*p* < 0.001) and mental health services (*p* < 0.001).

**Table 2:** Employment specialists: self-reported collaboration with clinical teams; self-reported collaboration with employers; and perception of IPS user’s health and well-being

Variable (during Covid-19 restrictions March-October 2020)	Mean	*p*-value
*Collaboration with clinical teams (n* = *25)*		
I have been integrated with my clinical team	1.96	< 0.001
I have been in contact with clinicians from my clinical team	2.04	< 0.001
The clinical team has appreciated the role of the job specialist	2.68	0.058
I have attended meetings with the clinical team	2.04	< 0.001
Clinicians have contacted me regarding patients on their patient list	2.28	< 0.001
I have kept records in the electronic record system	2.48	0.004
*Collaboration with employers (n* = *22)*		
I have been involved in job development	2.00	< 0.001
I have contacted employers directly	2.09	< 0.001
Employers have contacted me	2.23	< 0.001
I have organised job interviews for users	1.82	< 0.001
Users on my list want to apply for jobs	2.41	0.006
There are vacancies available	2.09	< 0.001
*Employment specialists perception of IPS user’s health and wellbeing (n* = *25)*		
Worried	3.80	< 0.001
Socially isolated	3.92	< 0.001
Their mental health has varied	3.76	< 0.001
They need more help from mental health	3.52	< 0.001
They're contacting me	2.92	0.574
They have needed my support	3.48	0.001
They receive support from me	3.48	< 0.001

(One sample *t*-tests based on Likert scales: Test-value = 3 (same as usual), 1 = much less than usual, 5 = much more than usual.)

We also compared the different forms of communication that employment specialists used for interacting with IPS users before and during Covid-19 restrictions (Table 3). There was a significant increase in the proportion of employment specialists, 4% versus 60%, who used video solutions (skype, Microsoft teams etc.) to communicate with IPS users (*p* < 0.001) (Table 3). Only 4% (1) of employment specialists indicated that they would continue using virtual communication platforms after Covid-19 restrictions end. Interestingly, there was no significant change in the proportion who had physical meetings with IPS users (Table 3).

**Table 3:** Modes of communication that employment specialists used to communicate with IPS users pre covid vs during covid. n = 25

Modes of communication	Before Covid-19 restrictions were introduced	During Covid-19 restrictions	McNemar's change test
	n (%)	n (%)	*p*-value
Email	10 (40)	14 (56)	0.13
Physical meeting	23 (92)	19 (76)	0.29
Mobile	25 (100)	25 (100)	1.00
Text message	23 (92)	22 (88)	1.00
Any video solution (i.e. Skype, Microsoft Teams)	1 (4)	15 (60)	< 0.001

## Discussion

### Context

IPS services and the employment specialist role have become increasingly important during the Covid-19 pandemic. When economies contract, people with mental disorders may be at higher risk of losing their jobs and more competitive labour markets can also make it more difficult to find a new job (Evans-Lacko et al., 2013). Therefore, measures such as IPS that help people with severe mental illness obtain and maintain employment are of fundamental importance (Drake et al., 2021).

Covid-19 containment measures have been inimical to IPS services in Norway at several levels. Firstly, IPS is doubly vulnerable to Covid-19 restrictions because it is reliant on extensive co-operation between two separate governmental bodies (NAV and Health Services). These services became less interconnected due to social distancing policies, weakening the employment specialists' integration in clinical teams. Secondly, business closures and decreased job vacancies have unfavourably impacted the employment specialists' collaboration with managers and employers in the open job market, thereby hindering the job search process for service users.

### Findings

Employment specialists in Northern Norway have continued to successfully provide IPS services during the Covid-19 restrictions between March and October 2020. Despite a sharp decrease in the number of job vacancies (Statistics Norway, 2021a), 20.4% IPS users on employment specialists lists obtained employment after Covid-19 restrictions were introduced in March 2020. Employment specialists reported that IPS users on their lists required more support than usual from both employment specialists and mental health services and that they did not want to apply for jobs as much as usual after the Covid-19 restrictions were introduced. This provides valuable evidence that IPS employment specialists have an important therapeutic role beyond job support; it appears that IPS users turn to their employment specialist for non-work-related support when they experience deterioration in their health and well-being. Positively however, employment specialists also indicated that around 82.8% of unemployed IPS users continued seeking competitive employment during Covid-19 restrictions, despite experiencing a decreased sense of well-being. The Norwegian government's strong social welfare and labour market Covid-19 protection strategies (National Insurance Act, 2020a, 2020b, 2020c; Norwegian Tax Administration, 2021), may have helped mitigate the effects of the pandemic on services users' ability and motivation to find employment.

In March 2020 many employment specialists were reassigned to other roles or received extra, non-IPS related work tasks as a result of resource shifting due to the Covid-19 response. Considering that IPS is relatively new and not fully implemented in Norway, service quality may have declined when employment specialists were expected to prioritise non-IPS related tasks. Employment specialists also reported less collaborative engagement with employers on the open job market and with clinical teams. However, it is encouraging that the IPS services continued to provide a service whereas other employment support services for people with mental health conditions and addictions in Norway closed during Covid-19 restrictions. Our findings raise concerns around the fidelity of IPS implementation in Northern Norway during Covid-19 restrictions. IPS programs utilise two fidelity scales to assess if IPS is being implemented as intended (Becker et al., 2015). Fidelity guidelines recommend that employment specialists participate in weekly meetings with clinical teams (Becker et al., 2015; Rinaldi and Perkins, 2007). In addition, they are expected to have ongoing communication and collaboration with clinical teams between meetings (Becker et al., 2015; Rinaldi and Perkins, 2007).

The Covid-19 restrictions have led to significant changes in the way people work, including increased use of virtual communication platforms for work-related meetings (Karl et al., 2021). Furthermore, the increased use of virtual communication and the concurrent decrease in-person meetings may continue long after the pandemic ends (Standaert et al., 2021). Mental health services have adjusted to the demands of Covid-19 accordingly (Moreno et al. 2020), illustrated by the rapid and widespread expansion of telepsychiatry (O'Brien et al., 2020). While virtual mental health services are particularly suitable during the pandemic, it is likely to remain a viable supplement to mental health care post Covid-19 (O'Brien et al., 2020).

Our study confirms that employment specialists in Northern Norway aptly adapted IPS to pandemic circumstances through swift, large-scale adoption of video solutions to communicate with IPS users. However, the majority (96%) do not think they will continue using virtual communication platforms after Covid-19 restrictions are lifted. This compelling finding highlights the fundamental importance of face-to-face contact for IPS, especially given that many employment specialists in Northern Norway need to make long and time-consuming commutes through sparsely populated areas in order to deliver IPS services. Avoiding the time and costs of commuting is identified as an important benefit of stay-at-home policies and many workers welcome the possibility of a permanent shift to remote work (RESPOND Project, 2021; Green et al., 2020).

Interestingly, there was no concurrent decrease in the proportion of employment specialists who had physical meetings with IPS users. This finding may reflect the fact that, compared to its Nordic neighbours and many other European countries, Norway responded quickly to the Covid-19 threat by implementing strong containment strategies in March 2020, thereby allowing Norway to have relatively lenient Covid-19 restrictions later in year (Ritchie et al., 2020).

The medium to long term impact of Covid-19 on the economy and in turn the impact on mental health services is unknown. Previous research has shown that periods of economic uncertainty can cause fluctuations in the availability of rehabilitation and recovery focused mental health services (Warner, 2004) and impacts on the employment rates of people with severe mental health problems (Rinaldi et al., 2011). However, ensuring people using mental health services can access the support to gain and retain employment during times of economic uncertainty becomes even more critical with calls for employment to be a treatment outcome for all psychiatrists (Greenberg et al., 2022).

### Originality

To our knowledge, no previous study has examined the impact of Covid-19 restrictions on IPS service delivery in the Norwegian context.

### Limitations

These results require cautious interpretation as the small sample size increases the risk of type-2 errors. Another limitation concerns generalisability, we have not established if the results of this study can be used to make inferences about the impact of Covid-19 restrictions on IPS service delivery in other contexts or whether the restrictions were the problem or the consequences of the restrictions. In addition, the study only looked at the experiences of the IPS employment specialists and not people using mental health services. Research from the USA shows that people with mental health conditions who had jobs, valued being able to continue working during the pandemic (Cook et al., 2022). Finally, the survey took place in October 2020 and covered a short time period (March-October 2020) where the level of restrictions was relatively low, responses may have varied between different periods in the pandemic and the effect may not have been maintained.

### Conclusion

Covid-19 restrictions appear to have created obstacles for IPS service delivery in Northern Norway. IPS employment specialists have been resilient and shown strong capabilities in overcoming these challenges. They have continued to successfully fulfil their main responsibilities such as helping IPS users gain and maintain competitive employment as well as meeting with IPS users to listen and provide support. However, there are concerns that Covid-19 restrictions have negatively impacted the employment specialists integration and collaboration with clinical teams which may become apparent in future implementation fidelity assessments.

## Data Availability

The datasets generated and analysed during the current study are not publicly available due to participant confidentiality but are available from the corresponding author on reasonable request.

## Abbreviations

IPS: Individual placement and support
NAV: The Norwegian labour and welfare administration
SMI: Severe mental illness
RCT: Randomised control trial
WHO: World Health Organisation
OECD: Organisation for economic co-operation and development
